# Effects of *Rhazya Stricta* plant organic extracts on human induced pluripotent stem cells derived neural stem cells

**DOI:** 10.1371/journal.pone.0288032

**Published:** 2023-07-21

**Authors:** Abdullah Othman Alawad, Faisal Sultan Alagrafi, Ahmed Jaman Alfahad, Hala Abdulrahman Alamari, Fatimah Othman Alghamdi, Hussam Mokhtar Fallatah, Alhassan Hamood Aodah, Sultan Suleiman Alyousef, Muhammed Adnan Bakhrebah, Ibrahim Oqla Alanazi, Mohannad Mokhtar Fallatah

**Affiliations:** 1 Aging Institute, Health Sector, King Abdulaziz City for Science and Technology, Riyadh, Saudi Arabia; 2 Bioengineering Institute, Health Sector, King Abdulaziz City for Science and Technology, Riyadh, Saudi Arabia; 3 Waste Management and Recycling Institute, Sustainability and Environment Sector, King Abdulaziz City for Science and Technology, Riyadh, Saudi Arabia; 4 Advanced Diagnostics and Therapeutics Technologies Institute, Health Sector, King Abdulaziz City for Science and Technology, Riyadh, Saudi Arabia; Adama Science and Technology University, ETHIOPIA

## Abstract

*Rhazya Stricta* (*R*. *stricta)* has been employed as a natural remedy for several diseases for centuries. Numerous studies revealed that *R*. *stricta* extracts contain alkaloids, tannins, and flavonoids that possess antimicrobial, anticancer, antihypertensive, and antioxidant activities. In this study, we examined the effects of organic extracts from different parts of *R*. *stricta* plant on human pluripotent stem cells (hiPSCs)-derived neural stem cells (NSCs) for medical purposes. NSCs were incubated with different concentrations of organic extracts from the leaves, stem, and fruits, and we assessed the growth and viability of the cells by using MTS assay and the chemical composition of the potential plant extract by using gas chromatography-mass spectrometry (GC/MS). Our results revealed that the methanolic extract from the stem increased NSCs growth significantly, particularly at a concentration of 25 μg/ml. GC/MS analysis was utilized to identify the potential compounds of the methanolic extract. In conclusion, our results demonstrated for the first time that methanolic stem extract of *R*. *stricta* contains compounds that can positively impact NSCs growth. These compounds can be further investigated to determine the potential bioactive compounds that can be used for research and medical purposes.

## 1. Introduction

Neural stem cells (NSCs) are known for their ability to self-renew and differentiate into any type of cells found in the central nervous system (CNS), such as neurons, glial cells, and astrocytes [1]. Due to these properties, NSCs are used as an alternative promising therapeutic approach to treat neurodegenerative diseases, including Alzheimer disease (AD), Parkinson’s and Huntington’s diseases. NSCs can be transplanted into the patient to reverse neural tissue damage and to recover neurological disorders [2, 3], or loaded with a specific drug to act as a delivery vehicles, delivering and releasing the drug specifically in the diseased area without affecting normal tissues [2]. However, despite the promising outcomes of NSC-based therapies in preclinical studies, major concerns still limit their success in clinical studies. For example, utilising embryonic stem cells differentiated into NSC for therapeutic purposes is still ethically concerning [4]. Furthermore, isolating NSC from primary tissues is extremely dangerous, and NSC transplantation from a healthy donor may result in rejection [5]. To overcome these obstacles, researchers developed a new strategy to generate NSCs from other sources. The most convenient and acceptable source of NSCs is induced pluripotent stem cells (iPSCs) [6]. Human iPSCs have been widely used in medical research for the following reasons: iPSCs can be generated from adult somatic cells that have been genetically reprogrammed to an embryonic stem (ES) cell-like state; the capacity to differentiate into many types of cell lineage; iPSCs provide a powerful platform for disease modeling and drug screening [7, 8].

Since time immemorial, people used natural products to treat their diseases [9]. Despite the development of pharmacological agents for the treatment of chronic diseases, the discovery of new medicinal plants compounds continued to flourish, encouraging scientists to isolate and investigate the bioactive compounds of a wide range of medicinal plants [9]. In the late 18th century, a synthetic drug from the active ingredient of analgesic herbal remedies was revealed. More recently, and with the advancement in compound isolation and separation techniques, many crude plant extracts have been isolated and tested on different human cell types, including NSCs, and they have shown different actions ranging from neuroprotection, cell survival, and increased cell proliferation [10–13]. This hastened the emergence and dominance of pharmaceutical industries [14]. Although numerous plant natural products have been remarkably shown to be effective treatments for neurodegenerative diseases, previously classified as non-curable diseases [15–17], there are still a large number of plants in ecologically diverse areas such as Saudi Arabia that have not yet been discovered for their potential therapeutic efficacy. The harsh environmental conditions in Saudi Arabia, such as heat and lack of water, force the plants to evolve and develop "survival molecules" and metabolites [18].

*Rhazya stricta* (*R*. *stricta*), or "Harmal" in Arabic, is a native evergreen dwarf poisonous shrub commonly found in the sandy plains of Saudi Arabia, the Middle East countries, India, and Pakistan. It is known for its multiple pharmacological actions and was traditionally used as a medicinal herb in different forms, fresh or dried, to cure a number of diseases such as stomach abdominal pain, sore throat, foot burning, cancer, diabetes, etc. [18, 19]. In addition, bioactive compounds of *R*. *stricta* have been extracted from different parts of the plant, and showed various biological activities, including antimicrobial, anticancer, and antioxidant [18, 20]. However, the effect of *R*. *stricta* extracts on NSC has not yet been investigated. Here, we extracted active ingredients from different parts of the plant using several organic solvents and tested their effectiveness on human iPSC-generated NSCs. We found that the methanolic extract maintained NSC viability and promoted their growth significantly. The GC/MS analysis of the corresponding extract revealed a predominant amount of quebrachmine, which may attributed to the biological activity of the crude extract.

## 2. Materials and methods

### 2.1 Plant material collection, extraction and isolation

*R*. *Stricta* plant materials were collected and processed as previously published by Alagrafi *et al*. [21]. Briefly, air dried *R*. *stricta* materials (fruits, leaves, and stem) were crushed into powder. Then, around 50g of each part was extracted with 500ml of each solvent, including Hexane, Chloroform (CHCl_3_), Ethyl acetate (EtOAc) and Methanol (MeOH) through a Soxhlet apparatus for 24h. Next, samples were centrifuged at 4000 rpm for 10 min to remove any residual. The supernatant was collected and concentrated in a rotary evaporator under low pressure at 45°C. Finally, the dried extracts were dissolved in MeOH. The following equations were used to calculate the amount of extract.


$$\%\text{Total}\;\text{extraction}\;\text{yield}=\left(\text{extract}\;\text{mass}/\text{sample}\;\text{mass}\right)\;\text{x}\;100$$

### 2.2 Gas Chromatography Mass Spectrometry (GC/MS) analysis

The extract was analysed on a gas chromatography-tandem mass spectrometry instrument (TSQ Quantum XLS, Thermo Scientific, USA). Chromatography separation was achieved using a GC-column, TR-35MS (30 m x 0.25 mm x 0.25 μm film thickness, Thermo Scientific). One microliter of diluted sample was injected in splitless mode, and the injector temperature was set at 220°C. The carrier gas was high-purity helium (99.999%) and the flow rate was 1.2 mL/min. The oven temperature was initially maintained at 60°C for 3 min, then increased to 200°C at 30°C/min, then to 300°C at 5°C/min, and held for 5 min (the total run time was 36.2 min). The transfer line temperature was 250°C. The conditions of the mass detector were as follows: ionisation mode, electron impact (EI) at 70 eV, full scan range 35–1000 m/z, the ion source temperature was 250°C. Natural compounds in all samples were processed using Xcalibur software and were identified using the NIST 14 database.

### 2.3. Cell viability and cytotoxicity assays

The colorimetric method from (Promega, G3581) was used to evaluate the biological activity of crude methanolic extract of *R*. *Stricta* stems (RSS MeOH) for the proliferation and survival of the generated NSC compared with its relevant vehicle control containing equal amounts of (MeOH). Cells were plated overnight at a concentration of 25,000 cells/well of a 96-well plate coated with geltrex. One day after seeding, the cells were treated for 48 hours with serially diluted concentrations of (RSS MeOH) ranging from (0.09 to 100 μg/mL), or with equal amount of vehicle control (MeOH). After the treatment, MTS solution was added at a concentration of 1:5 to the culture media, then cells were incubated for 2 hours. After the incubation, the plates were read at 490 nm. Wells containing media only without cells were used as a blank for background correction. The statistical analysis of all data was performed between the treated group with (RSS MeOH) and its solvent methanol as vehicle control for each concentration versus untreated cells (control). The percentage of cell viability was calculated for the untreated cells (control) as given in the formula below:

The cell viability percentage is calculated as (OD of treated minus OD of blank)/(OD of control minus OD of blank)x100.

### 2.4. Neural induction

High-quality hiPSCs were cultured in feeder-free conditions with Essential 8 medium on vitronectin up to 70–80%. Cells were detached by adding 0.5 mM EDTA to obtain cell clumps. Cell clumps were then plated at a density of 300x10^5^ per well in a 6-well plate containing fresh Geltrex with E8 medium and 10 M Rock inhibitor. On day 1 post-culturing, iPSCs were 15 to 20% confluent. E8 medium was replaced with neural induction medium (Thermo life technologies) to remove non-attached cells and Rock inhibitor. The differentiating cells were maintained in a neural induction medium for 7 days, and the media was changed every two days. On day 8 post culturing, the differentiated cells were then harvested and expanded. Next, cells were detached by adding accutase to the cells then collected by centrifugation. Cells were cultured at 0.5x10^5^ cells/cm2 in Geltrex with complete Neural Expansion Medium and 5M Rock inhibitor, then incubated overnight. The media was replenished to remove the ROCK inhibitor. The generated NSCs were passaged every 4–6 days with accutase (Thermo Fisher Scientific).

### 2.5. Characterisation of Neural Stem Cells (NSCs)

#### 2.5.1. Polymerase Chain Reaction (PCR)

Total RNA from hiPSC or NSCs was extracted by using the RNeasy micro kit (Qiagen). Then, cDNA was prepared from the total RNA (1 μg) according to the Reverse Transcription kit instructions (Promega). PCR reactions were performed in a final volume of 25 μl by adding 12.5 μl of GoTaq^®^ Green Master Mix (Promega), 2.5 μl of cDNA (an equal concentration was used from each sample), and 1μl of each forward and reverse primers (5 pmol). Nuclease-free water was added to adjust the volume. Next, PCR reactions were amplified in the ProFlex PCR system (Applied Biosystems, USA) with a different number of cycles and annealing temperature depending on the primers (Table 1). PCR conditions were programmed as follows: initial denaturation at 94°C (5 min) followed by cycles of amplifications: 30 sec at 94°C, 30 sec at 53–61°C, 40 sec at 72°C, and final extension at 72°C for 10 min. PCR products and 100 bp DNA ladder were separated in 1.2% agarose gel containing SYBR Safe. The primer sequences of Oct4, Sox2, and cMyc were obtained from the hiPSCs protocol of Cambridge Biomedical Research Centre [22], and other primers were designed by using Primer-BLAST at GeneBank website (http://www.ncbi.nlm.nih.gov/tools/primer-blast/). All the primers were purchased from Humanizing Genomics Macrogen, South Korea, and then they were dissolved in 1x TE buffer and stored at– 20°C until use. The PCR amplification was carried out in two independent experiments. The gene expression of PCR products was normalised to h-β. Actin as an internal control to distinguish the gene expression profile of NSCs post the differentiation compared to iPSC as a negative control.

**Table 1:** List of primers and PCR conditions used for this study.

Primer	sequence	Product size (bp)	Annealing Tem	No of Cycles	Reference
h-Oct4	F-CCTCACTTCACTGCACTTGTA R-CAGGTTTTCTTTCCCTAGCT	165 bp	55	32	(BRC, 2014)
h-Sox2	F- ATGTCCCAGCACTACCAGAG R- GCACCCCTCCCATTTCCC	141 bp	55	29	(BRC, 2014)
h-Myc	F-CTGAAGAGGACTTGTTGCGGAAAC R- TCTCAAGACTCAGCCAAGGTTGTG	190 bp	55	29	(BRC, 2014)
h-Kf4	F- GGTCGGACCACCTCGCCTTACAC R- CTCAGTTGGGAACTTGACCA	172 bp	55	30	(BRC, 2014)
h-Nanog	F- TTTGTGGGCCTGAAGAAAACT R- AGGGCTGTCCTGAATAAGCAG	116 bp	53	32	This study
h-β.actin	F- AAACTGGAACGGTGAAGGTG R- AGAGAAGTGGGGTGGCTTTT	171 bp	60	22	This study
h-Nestin	F- AGC CCTGAC CAC TCC AGT TTA G R- CCC TCT ATG GCT GTT TCT TTC TCT	128 bp	55	30	This study

#### 2.5.2. Immunocytochemistry (ICC)

To assess the protein expression levels, cells were fixed with 4% paraformaldehyde for 20 min at room temperature. Fixed cells were then washed twice with PBS and permeabilised in PBS with 0.2% Triton X-100 for 20 min (Amresco, OH, USA). Cells were blocked for 30 min in PBS with 5% normal goat serum (Millipore, CA, USA) and with 0.2% Triton-x-100, followed by overnight incubation at 4°C with the primary antibodies: anti-Nestin antibody (abcam, ab22035) and anti-sox1 antibody (abcam, ab109290). After the incubation, the cells were rinsed with PBS and incubated with secondary antibodies Alexa Fluor 488-conjugated goat anti mouse and Alexa Fluor 488-conjugated goat anti rabbit on a shaker for 30 min at room temperature. Subsequently, cells were rinsed in PBS and 0.5 μg /ml Hoechst (sigma) was added and incubated for 5 min to stain the nuclei. An inverse fluorescence microscope was used for cell imaging.

### 2.6. Statistics

The results are presented as the mean ± standard deviation (± SD) of two independent experiments, and statistical analysis was performed using Student’s t-tests. *p*-value less than 0.05 was considered statistically significant.

### 2.7. Ethical approval

This study involved the collection of plant. This work was permitted by the Health and Life Science Research Institute.

## 3. Results

### 3.1. Induction of neural stem cells from human pluripotent stem cells

**Fig 1:**
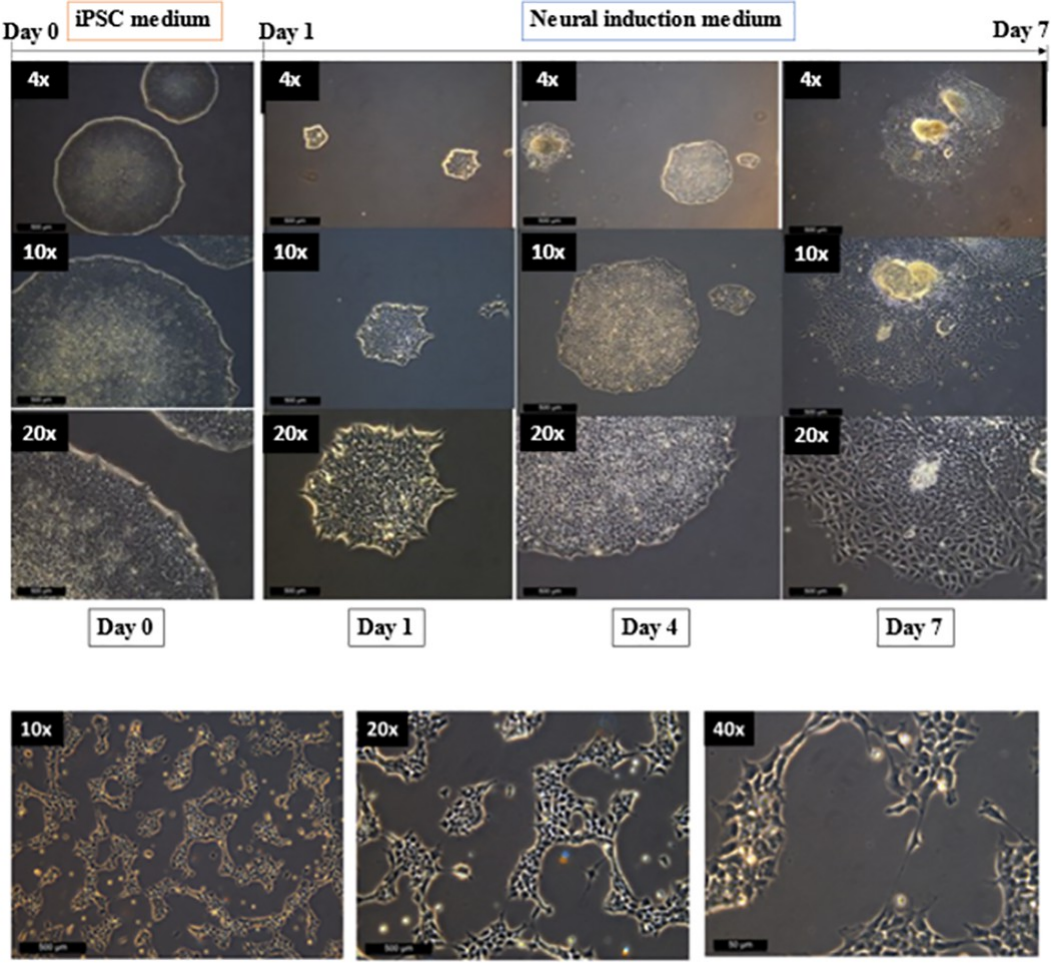
(a) Timeline of induction of NSCs from human iPSCs (hiPSCs). (b) Days 1–7 show the initiation and the process of neural induction from the colonies. Clear morphological changes of NSCs differentiated from hiPSCs *In vitro*, cells became elongated. This image was taken from passage 2.

To study the effect of *R*. *stricta* plant extracts on NSC, we have first generated functional NSCs from non-differentiating hiPSCs by culturing them in PSC neural induction medium for seven days. During the neural induction process, hiPSCs-NSC induced cells started to proliferate, and their density increased (Fig 1a). Induced NSCs were then harvested on day eight and expanded in an expansion medium and they were passaged every 4–6 days (Fig 1b).

### 3.2. Characterisation of the generated NSCs

**Fig 2:**
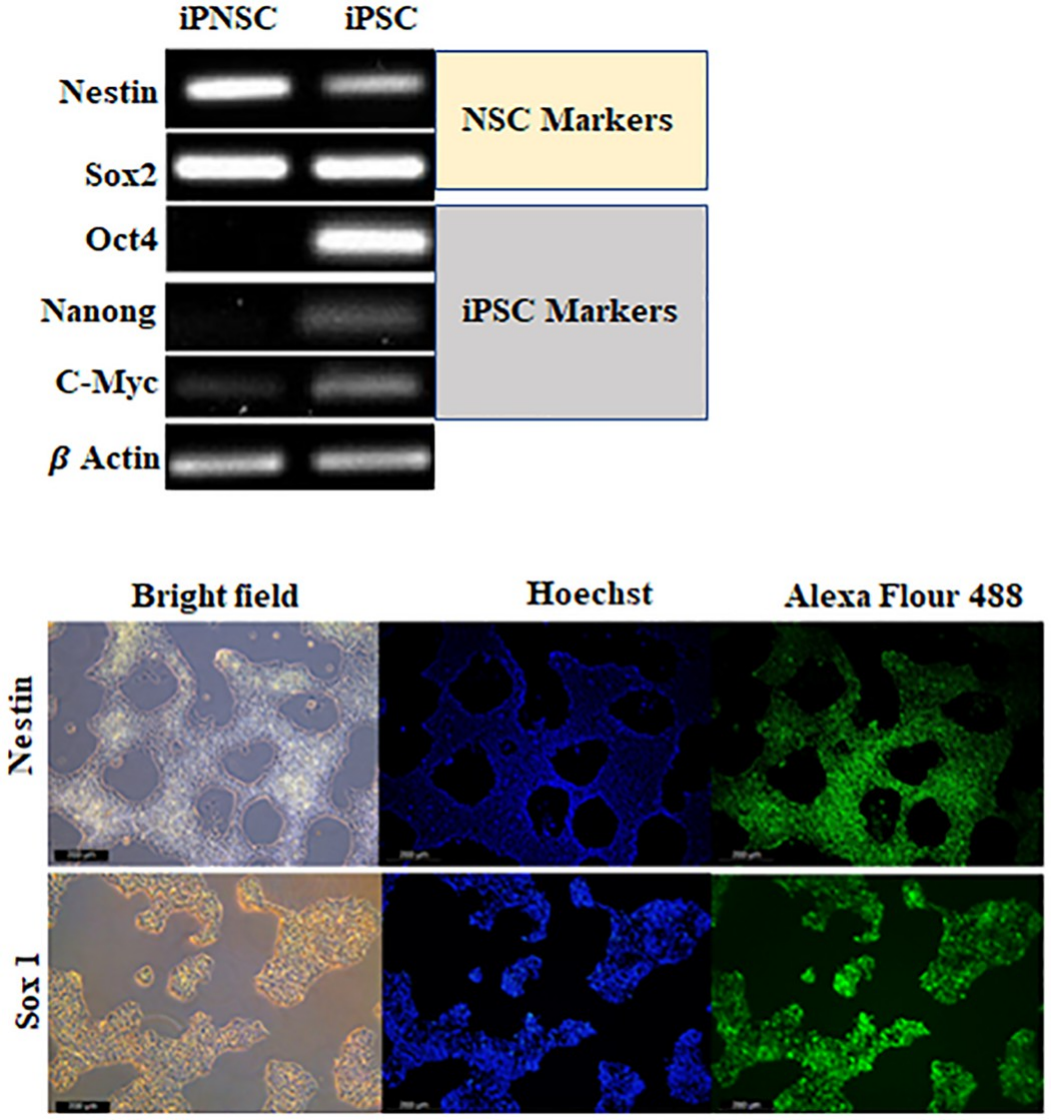
(a) PCR analysis of expression pattern of NSC markers (Nestin and Sox2) and the downregulation of iPSCs markers (Oct4, Nanog and C-Myc) post the differentiation. (b) NSCs derived from human iPSCs stained with antibodies (Alexa Fluor 488) against the neural stem cell markers Nestin (upper panel) and Sox1 (lower pannel). Nuclei was stained by Hoechst. (Magnification 10x).

In order to confirm the conversion of hiPSC into NSCs, we have tested a list of transcription factors by PCR (Table 1), in both hiPSC-generated NSCs and in the hiPSC as a negative control. The differentiation of hiPSCs into NSCs was confirmed by the lack of Oct4 and nanog expressoin on day 7 post NSC induction, accompanied by a significant reduction of C-myc gene (Fig 2a), which goes in line with other studies where C-myc developmentally downregulated the downstream of Oct4. Also, this can be attributed to heterogeneity of the differentiated hiPSCs. Sox2 was highly expressed on the generated NSC as well as on iPSCs population, confirming the pluripotent state of the untreated cells and the neural lineage generation of the differentiated hiPSCs. Neural differentiation was also confirmed by the expression of Nestine. Untreated cells showed a faint band of this marker (Fig 2a), which is known as an early ectoderm marker [23], and this might suggest spontaneous differentiation event that took place in the untreated cells. Low gene expression in either cell population can also be attributed to the concentration of the cDNA while endpoint PCR was used. This could be further clarified by using RT-PCR.

To further confirm NSC generation from the hiPSCs, we have analysed the protein expression levels of Sox2 and Nestin on the generated NSCs by the Immunohistochemistry technique. iPSC- converted NSCs were prepared and stained by Abs against the specific NSC antigens Sox1 and Nestin. The nuclei were stained with Hoechst. Cells were then observed under the fluorescent microscope. The stained NSCs expressed both markers, Sox1 and Nestin as shown in (Fig 2b). Collectively, these results indicated that iPSC has been successfully converted into NSCs.

### 3.3. The yield of Soxhlet extraction

**Fig 3:**
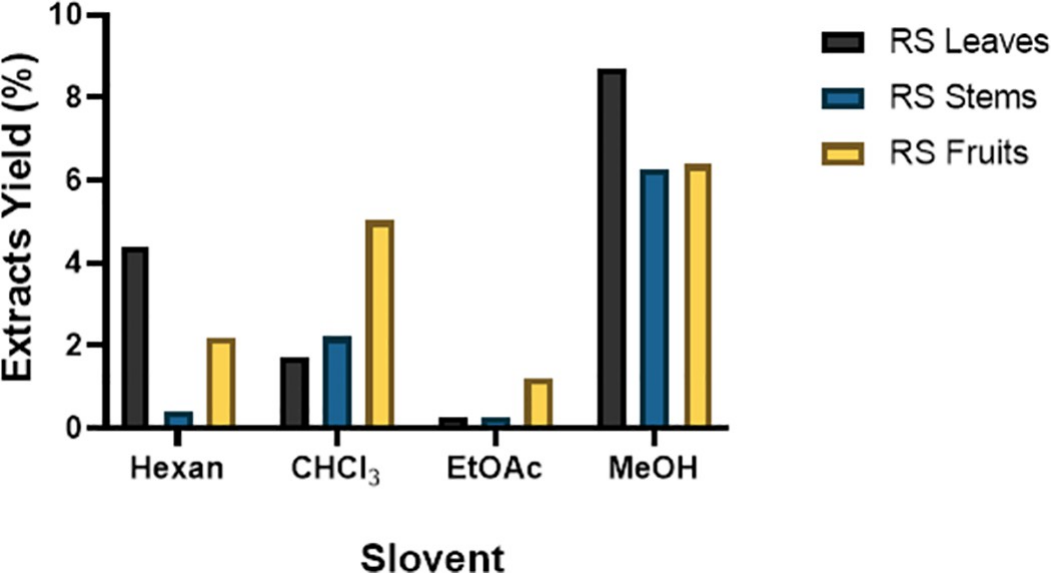
Polarity and the yield of Soxhlet extraction.

The yield of the extract was obtained from a dried powder (50g) from different parts of the plant, including the stem, leaves and fruit of *R*. *stricta* by using different solvents: hexane, CHCl_3_, EtOAc and (MeOH). The highest yield of extraction was obtained from the leaves with MeOH solvent (8.7%) followed by the extract from the fruit and stem (6.38% and 6.26%) respectively (Fig 3). However, the lowest yield of extraction was collected from the EtOAc (Fig 3). An almost undetectable amount of plant extract was collected from the leaves and the stem, and only (~1%) was collected from the fruit (Fig 3).

### 3.4. The effect of *R*. *Stricta* extracts on NSCs

**Fig 4:**
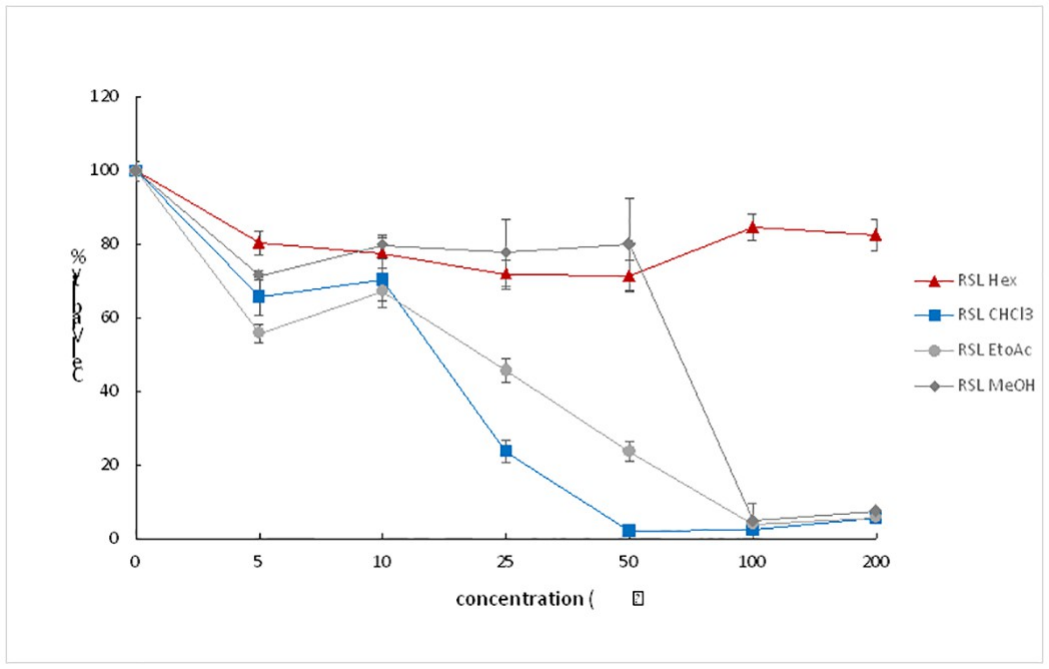
This graph shows the percentage of NSCs viability after 48h treatment with different concentrations of *Rhazya Stricta* leaves (RSL) extracts.

**Fig 5:**
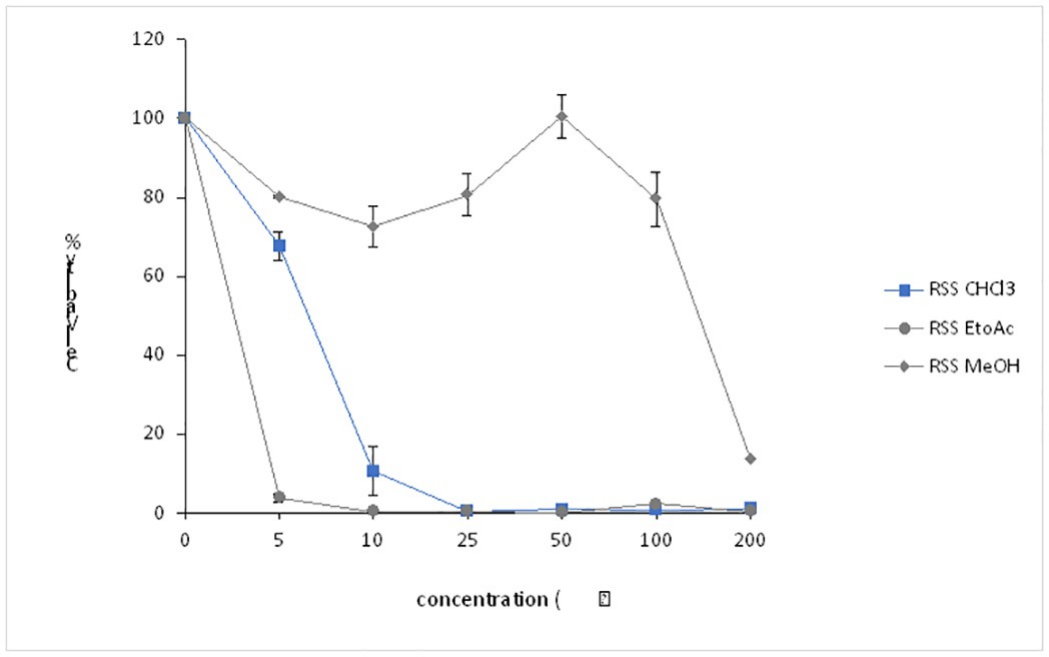
This graph shows the percentage of NSCs viability after 48h treatment with different concentrations of *Rhazya Stricta* stem (RSS) extracts.

**Fig 6:**
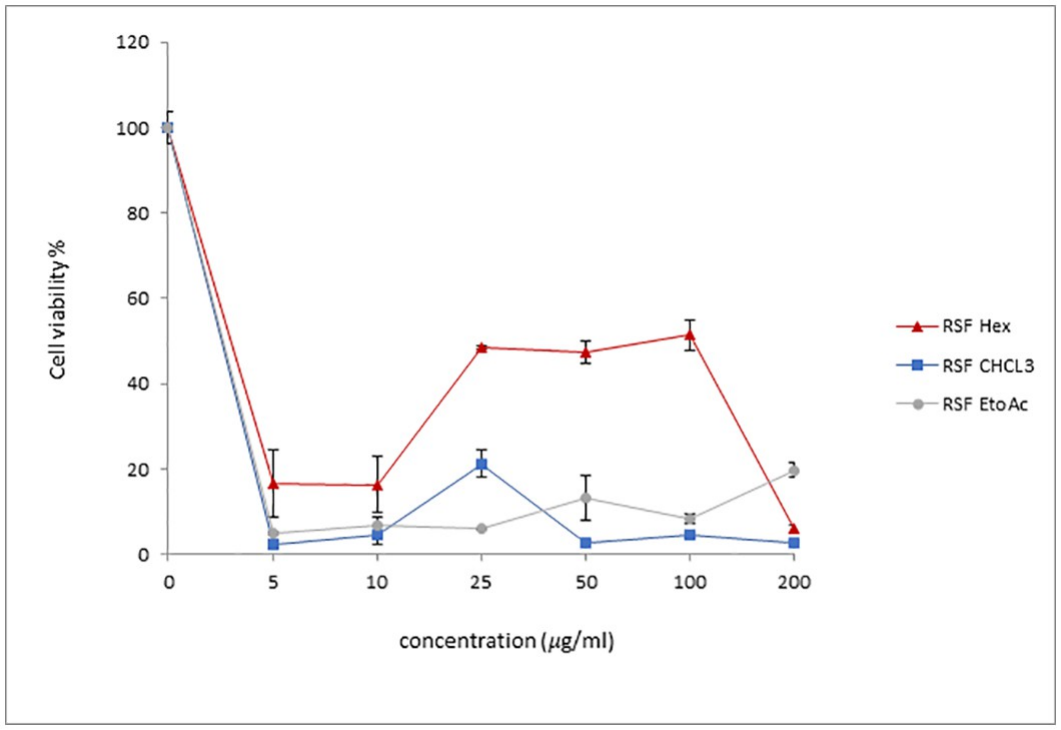
This graph shows the percentage of NSCs viability after 48h treatment with different concentrations of of *Rhazya Stricta* fruit (RSF) extracts.

To investigate the effect of *R*. *Stricta* extracts on hiPSC-NSCs viability, NSCs were seeded at 25.000 cells/well of 96 well plates overnight. Next, the plant extract was added to the wells at different concentrations starting from 5 to 200 μg/ml for 48 hours. Finally, MTS solution was added to the cells prior to reading the absorbance. The results showed that the untreated samples were 100% viable. Meanwhile, the presence of the organic solvent decreased NSCs viability at different ratios (Figs 4–6). The *R*.*stricta* leaves (RSL) Hex extract showed some protective properties for the treated NSCs. Their viability was between 70–80% at the following concentrations (5–50μg/ml) (Fig 4). NSCs viability was enhanced and reached (~80%) vaibility with the concentrations (100 and 200μg/ml) (Fig 4). Similar results were obtained when the cells were treated with (5–50 μg/ml) of RSL MeOH extract (Fig 4). However, NSCs did not become viable entirely at the highest concentrations (100 and 200μg/ml). The cell death protective effect of the RSL was abolished with both EtOAc and CHCl_3_, NSC viability notably decreased with higher concentrations of EtOAc and CHCl_3_ (Fig 4).

NSCs viability results were significantly variable with RSS extracts. The percentage of viable cells was about 65% in the precence of (5μg/ml) of CHCl_3_, and droped to 10% with (10μg/ml) of the same solvent, before they completely lose their viability with higher concenterations (25–200μg/ml) (Fig 5). Notably, the percentage of viable NSCs was almost undetectable with EtOAc solvent, even when cells were incubated with very low pwercentage (5μg/ml) of the solvent (Fig 5). However, the results were different with RSS MeOH extract. The plant extract inhibited NSC death when cells were treated with low concentrations 5 and 25μg/ml of RSS MeOH, resulting in 70 to 80% of the NSCs being viable respectively (Fig 5). Interestingly, incubating the converted NSCs with 50 μg/ml of the RSS MeOH extract resulted in complete protection from cell death, as 100% of the treated NSCs were viable (Fig 5).

In contrast to RSL and RSS, *R*. *Stricta* friut (RSF) extracts effect on NSCs was modest. NSCs viability dropped drastically when the cells were treated with low concentrations (5 and 15 μg /ml) of the organic solvents (between 5–20%) (Fig 6). These percentages of cell viability remained almost the same when higher concentrations of CHCl_3_ and EtOAc were used (Fig 6). However, some protection capability was observed with RSF Hex extract, particularly between 25 and 100μg/ml, such that approximately 50% of the RSF Hex extract treated NSCs were viable (Fig 6). The protection capability for the RSF Hex extract was ineffective with the high concentration (200μg/ml). Collectively, these results indicated that *R*. *Stricta* plant extract particulary from the stem part can protect NSCs from the toxicity associated with the organic solvent, and that the potency of the protection depends on the source of the extract. These findings also imply that RSS extract may provide complete protection for RSS MeOH treated NSCs.

### 3.5. RSS MeOH crude extract gave NSCs a growth advantage

**Fig 7:**
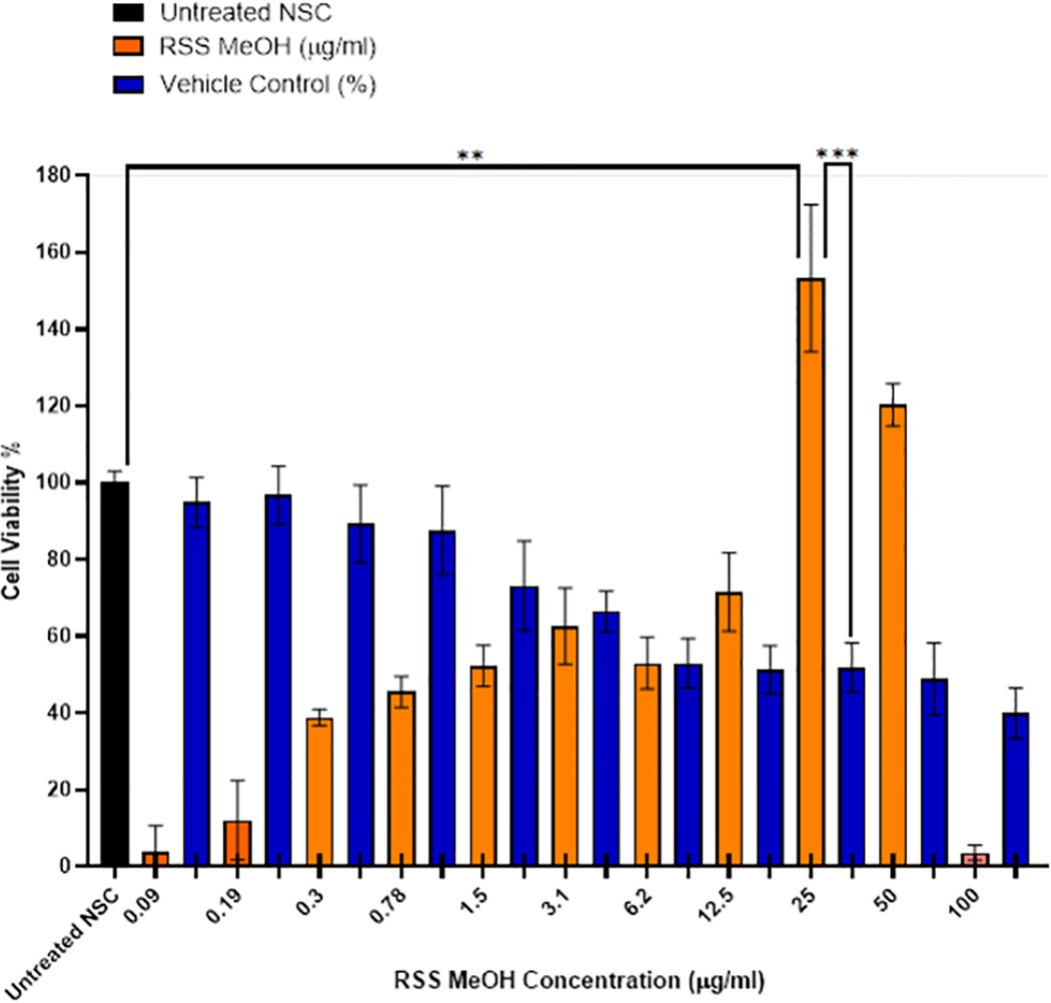
Equal number of NSCs (25,000 cells) were left without treatment, treated with vehicle only or with different concentrations of RSS MeOH extract.

After the encouraging results that were obtained from treating NSCs with RSS extracts, we then further investigated the biological activity of the crude RSS MeOH extract, which gave the best results on the NSCs out of the other solvents (Fig 5). Briefly, 25,000 hiPSCs-converted NSCs were plated in a 96 well plate overnight, then cells were treated with different concentrations of RSS or left without treatment as a control. We noticed that NSCs lose their viability gradually when they were exposed to a higher concentration of the vehicle (MeOH), until it reached ~40% of the treated cells being viable (Fig 7). However, in the presence of RSS extract, NSCs retained their viability. Interestingly, at 25μg/ml of RSS MeOH-extract, we found that NSCs growth increased significantly and reached 150% compared with the untreated group and the group treated with MeOH (100% and ~50% respectively) (Fig 7). These findings suggested that applying 25μg/ml of RSS MeOH extract protected NSC from MeOH toxicity and promote their growth significantly.

### 3.6. Gas chromatography–mass spectrometry analysis

**Fig 8:**
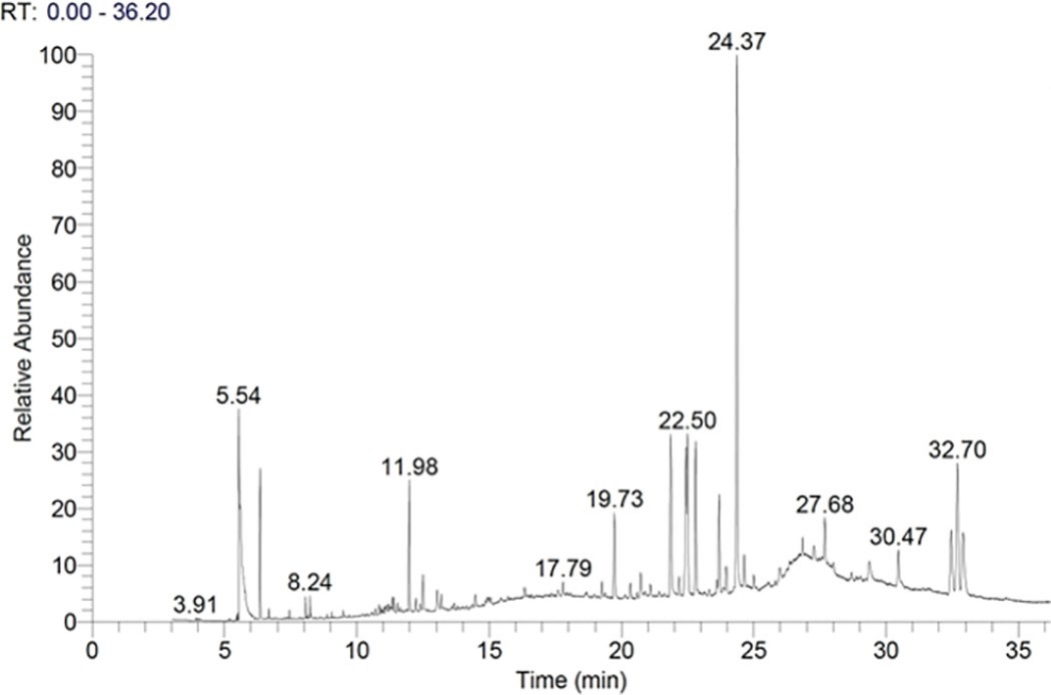
GC/MS chromatogram analysis of the methanolic extract of RSS.

**Fig 9:**
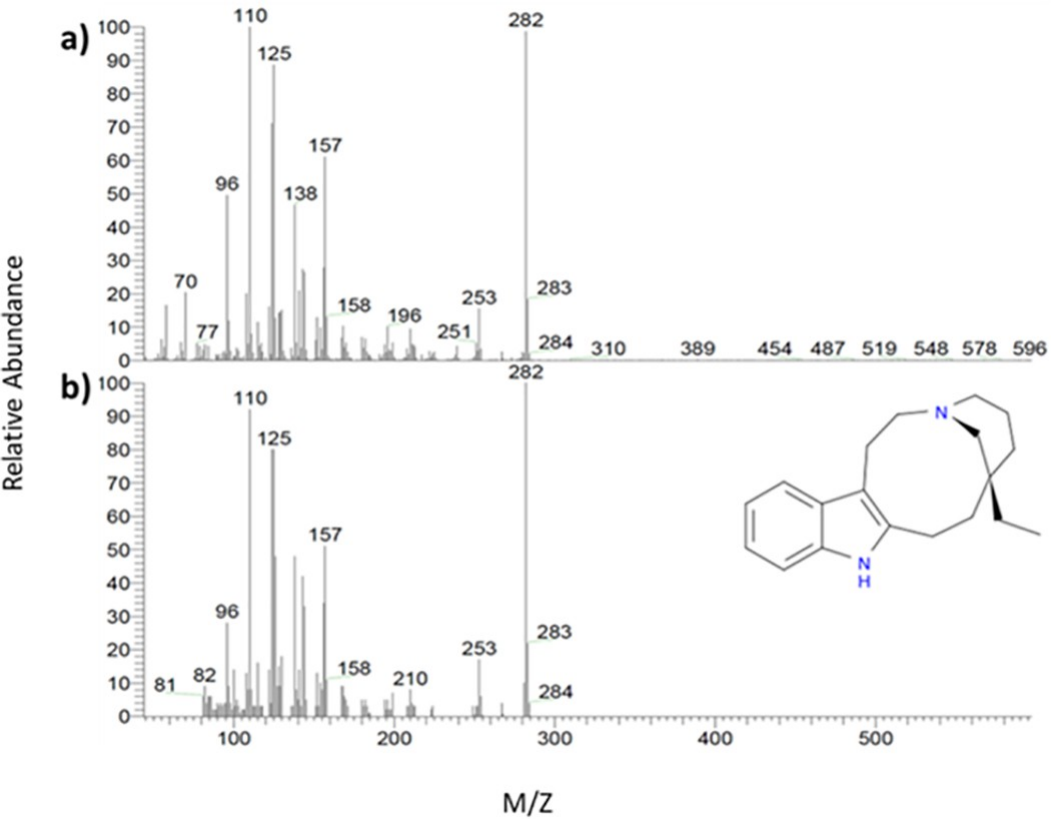
Comparison spectra between: a) The EI spectra of the compound at Rt = 24.36, and b) The NIST mass spectral of quebrachamine with (97% probability).

Gas chromatography–mass spectrometry (GC/MS) analysis was used to identify the composition of the methanolic extract. The GC-MS chromatogram showed many compounds (S1 File), but quebrachamine was predominant (Figs 8 and 9). The identification of quebrachamine was based on the great matching of EI spectra of the compound with NIST mass spectral (97% probability) (Figs 8 and 9).

## 4. Discussion

*R*. *Stricta* plant extracts and the isolated compounds have predominant anticancer and antibacterial properties [24, 25]. Here, we showed for the first time that RSS extract can also promote NSCs growth. We first generated NSCs *in vitro* from hiPSCs (Figs 1 and 2). The generation of NSC was confirmed by their appearance and by the expression of the three NSC markers [26–31]. NSCs had neurite outgrowth, and they were positive for the NSC protein markers Nestin, SOX1 and SOX2 (Figs 1 and 2). On the contrary, NSCs expressed very low or undetectable levels of the following markers: Oct4, Nanong and c-Myc (Fig 2A). These results were similar to the previous studies where they showed the expression of NSC markers in matured NSCs and NSCs derived from either hiPSCs or hESCs [29, 30].

To determine which crude extract would protect hiPSC-NSC from cell death, we extracted the crude product from different parts of the *R*. *stricta* plant by using different solvents (Fig 3), and performing a cell viability assay. Interestingly, RSS extract protected NSCs from MeOH toxicity, and it was more potent compared to leaf and fruit extracts, particularly at 25μg/ml (Figs 4–6). These variations in the results could be due to the differences in the structural chemical composition of the plant parts and their abundance [32–34], resulting in different outcomes as a consequence and affecting the final results.

Next, we further investigated the biological activity of the crud extract crude on NSCs’ fate. Cells were incubated with low concentrations of MeOH-RSS extract ranging from (0–100μg/ml) for 48 hours, then we evaluated cell viability and growth. The results of the MTS assay showed that MeOH was toxic to the cells, as it was determined by decreased NSC viability when they were exposed to absolute MeOH in a dose-dependent manner (Fig 7). NSC viability was reduced by 50% compared to the untreated cells (Fig 7). However, adding 25μg/ml of MeOH-RSS extract abolished the MeOH negative effect, maintained cell survival, and promoted their growth significantly compared to MeOH-treated NSCs (Fig 7). This result supported the feature of neuroprotection for phytochemicals [10, 35]. For example, Kim and his colleagues demonstrated that treating NSC with Kuwanon V (KWV), a crude extract from the root of the mulberry tree (Morus bombycis), at 0.25, 0.5, 1.0, or 2.5 M increased NSC viability compared to DMSO-treated control cells [10].

The promising outcomes of RSS extract on NSCs encouraged us to fractionate and characterise MeOH-RSS crude extract to identify the bioactive compound. Our GC/MS chromatogram analysis showed that quebrachamine indol alkaloid is the most dominant compound in the extract (Figs 8 and 9). Data collected from previous studies reported that indol alkaloids have a neuroprotective property [36–38]. Yang X *et al*. have isolated indole alkaloids from a crude leaf extract of the Asain tree *Alstonia scholaris* and evaluated their effect on adult mouse NSC. Using a colorimetric assay, they found that indole alkaloids enhanced NSC survival and increased their proliferation significantly in a dose-dependent manner [39]. These results supported the key role of indol alkaloids found in RSS crude extract for NSC protection and the induction of their cell growth. However, further studies are required to verify whether the observed effects are due to quebrachamine alone or a synergy with another compound.

Identifying the bioactive component of a natural product is important for developing new drugs, particularly for treating neurodegenerative diseases. Today, several promising pure plant-derived alkaloids that have neuroprotective effects have been synthesised, hoping to be used as natural drugs to treat CNS diseases [40–46]. For instance, Alstoscholarisine indol alkaloid, known for its significant bioactivity in promoting adult NSC proliferation, has attracted different research groups to synthesis a pure form of it for medical purposes [44–46]. Thus, our encouraging data strongly suggests the isolation of pure quebrachamine and investigating its biological activity and toxicity. This pure compound might then be synthesized and used as a promising alternative for conventional therapies to treat neurodegenerative diseases effectively with no or low toxicity. However, safety concerns have been observed with some of the isolated alkaloids [43].

## 5. Conclusions

In this study, we have investigated the effect of *R*. *Stricta* on hiPSCs-generated NSCs. First, we have successfully generated NSCs from hiPSCs by using pluripotent stems cells (PSCs) neural induction medium. The generation of NSCs was confirmed by their morphology after day 7 post neural induction, and by their expression of NSCs markers Nestin, Sox 1 and Sox 2, and the down regulation of hiPSCs markers Oct4, Nanog and C-Myc. showed that 25μg/ml of MeOH-RSS extract protected NSCs from cell death and induced their growth significantly. Our GC analysis of MeOH-RSS crude extract indicated that the bioactive compound is most likely to be quebrachamine indole alkaloid. These promising results may encourage researchers to examine a pure form of quebrachamine from RSS part, as a potential drug for treating neurovegetative diseases.

## Supporting information

S1 File Supporting information contains all of the supporting tables. (PDF)

S1 Raw images (PDF)
