# Supplementary material for: Effects of Rhazya Stricta plant organic extracts on human induced pluripotent stem cells derived neural stem cells
Source: PLoS One. 2023 Jul 21;18(7):e0288032. doi: 10.1371/journal.pone.0288032 (PMC10361509; doi:10.1371/journal.pone.0288032)
Supplement: S1 File — (PDF) [file pone.0288032.s001.pdf]

The table shows cell counts used in the experiments and extracts concentrations from different parts of *Rhazya Stricta* plants including the leaves, fruits and stems.

**Extract concentration (Leaves)**	**Cell counts**
Hex	
5	201.100
10	194.420
25	179.900
50	178.400
100	211.600
200	206.600
**$CHCl_3$**	
5	164.700
10	176.000
25	95.225
50	5000
100	6325
200	14350
**EtoAc**	
5	139.850
10	16.837
25	114.475
50	59.400
100	10.000
200	14.500
MeOH	
5	17.922
10	199.750
25	194.750
50	200.110
100	12.000
200	18.500

**Extract concentration (Fruits)**	**Cell counts**
Hex	
5	41.225
10	41,027
25	121.172
50	118.825
100	128.400
200	14.875
**$CHCl_3$**	
5	5.725
10	22.050
25	52.925
50	6.802
100	11.052
200	7000
**EtoAc**	

5	41.225
10	41.027
25	121.150
50	118.820
100	128.400
200	14.875

**Extract concentration (Stem)**	**Cell counts**
MeOH	
5	200.400
10	181.400
25	201.450
50	250.025
100	198.850
200	34.375
**$CHCl_3$**	
5	169.300
10	26.775
25	1.552
50	2.400
100	1.900
200	3.100
**EtoAc**	
5	9.675
10	1.207
25	1.207
50	500
100	6.200
200	1.375

Cell Number following hiPSC-NSCs treatment with different concentrations of RSS extract

**RSS extract concentration**	**Cell counts**
0	250.000
0.09	9.500
0.19	30.200
0.3	97.150
0.78	113.825
1.5	130.575
3.1	156.525
6.2	132.500
12.5	178.780
25	383.165
50	300.625
100	8.825
