# Supplementary material for: Effects of Rhazya Stricta plant organic extracts on human induced pluripotent stem cells derived neural stem cells
Source: PLoS One. 2023 Jul 21;18(7):e0288032. doi: 10.1371/journal.pone.0288032 (PMC10361509; doi:10.1371/journal.pone.0288032)

S1_raw_images

Gel figures for NSC markers following total RNA extraction from hiPSC or NSCs. Molecular weight markers (100 bp DNA Ladder), 1st New England biolabs (Cat NO # N3231L), 2nd Promega (Cat No # G210A). Images were captured by Gel Doc XR+ system (Bio Rad, USA).

NSC and ip OCT4 NANOG MYC SOX1

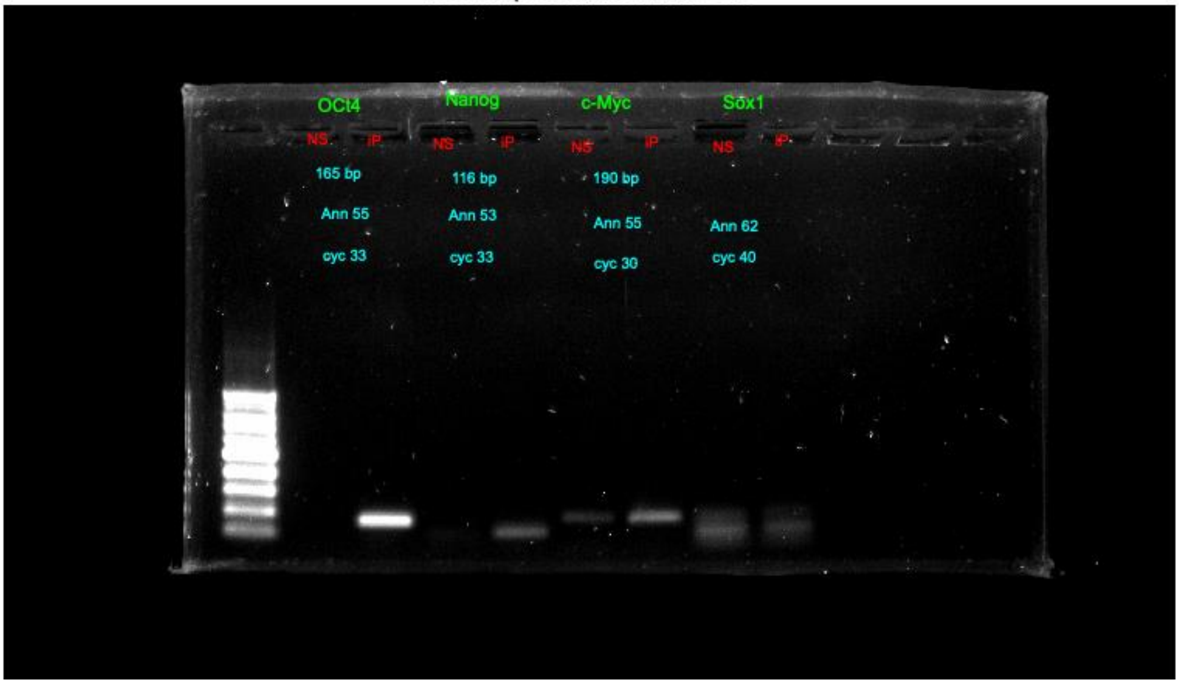

NSC and iP B.actin nestin sox2 sox1

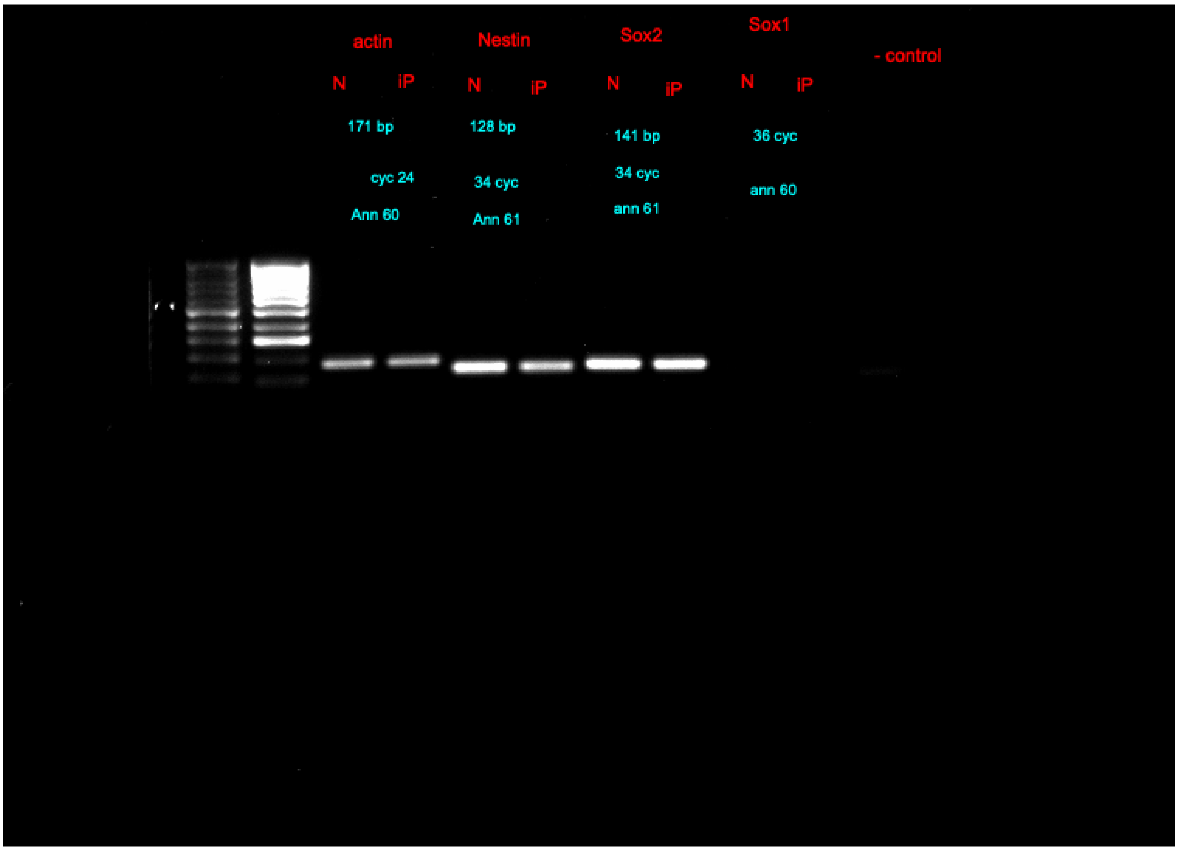

Supplement: S1 Raw images — (PDF) [file pone.0288032.s002.pdf]
